# P300 as an index of speech-in-noise understanding in complex acoustic environments in young and older adults

**DOI:** 10.3389/fnins.2025.1497781

**Published:** 2025-02-19

**Authors:** Dylan V. Pearson, Yi Shen, William P. Hetrick, Brian F. O’Donnell, Nancy B. Lundin, J. Devin McAuley, Gary R. Kidd

**Affiliations:** ^1^Department of Speech, Language, and Hearing Sciences, Indiana University, Bloomington, IN, United States; ^2^Department of Speech and Hearing Sciences, University of Washington, Seattle, WA, United States; ^3^Department of Psychological and Brain Sciences, Indiana University, Bloomington, IN, United States; ^4^Department of Psychiatry and Behavioral Health, The Ohio State University, Columbus, OH, United States; ^5^Department of Psychology, Michigan State University, East Lansing, MI, United States

**Keywords:** speech rhythm, aging, P300, speech-in-noise (SIN) perception, speech entrainment

## Abstract

**Introduction:**

Aging is associated with decrements in speech-in-noise perception which make communication in real world environments difficult. However, the neural correlates of these difficulties are not well characterized and finding correlations between speech-in-noise performance and electrophysiological measures has been difficult due in part to the fact that speech-in-noise perception is a multi-faceted process. The current study used a wide range of speech-in-noise tasks in an attempt to more completely capture speech-in-noise performance and compared these with electrophysiological measures.

**Methods:**

P300 event related brain responses were elicited in young and older adult listeners to spoken isochronous syllable sequences presented in quiet and noisy (i.e., multi-talker babble) background conditions. To investigate the extent to which P300 responses are associated with speech-in-noise understanding, listeners also completed a separate battery of speech-in-noise recognition tasks.

**Results:**

Overall P300 amplitudes measured in noisy conditions, but not in quiet conditions, were associated with performance on a variety of speech recognition tasks and were positively correlated with a composite measure of speech understanding in noise based on the full battery. In addition, older adults had P300 responses to deviant and omitted speech stimuli with lower amplitudes, longer latencies, and relatively greater frontal topographies than young adults.

**Discussion:**

The results demonstrate that P300 amplitudes elicited in competing noise were a strong predictor of speech-in-noise understanding. This, in addition to the age-related differences in P300 responses, highlights the relevance of neural attentional mechanisms for understanding age-related differences in speech understanding in noise.

## 1 Introduction

Difficulty understanding speech in noisy environments is a common problem reported by older listeners. This problem may be partially explained by the increasing rate of hearing impairment among older listeners (World Health Organization, 2018); however, speech-in-noise comprehension difficulty is prevalent among both older listeners with hearing impairments and those with normal hearing (Dubno et al., 1984; Summers and Molis, 2004). Many studies have investigated hearing-matched older and young listener groups and repeatedly found that audibility differences do not fully account for differences in speech-in-noise performance (Gordon-Salant and Fitzgibbons, 1993; Humes et al., 1994; Pichora-Fuller et al., 1995; Frisina and Frisina, 1997; Humes, 2021). Moreover, it is unclear which of the myriad of cognitive factors known to decline with age may best account for age-related decreases in speech-in-noise performance not accounted for by hearing impairment. For example, temporal processing (Konkle et al., 1977; Gordon-Salant and Fitzgibbons, 1993; Gordon-Salant and Fitzgibbons, 2004), working memory and executive function (Baddeley, 2002; Verhaeghen and Cerella, 2002), and processing speed (Salthouse, 1996) all show age-related declines, but the relation between these factors and speech-in-noise understanding is not fully understood.

Investigating whether neural correlates of age-related cognitive decline in older adults (Sharp et al., 2006; Shafto et al., 2007; Newsome et al., 2013; Rienäcker et al., 2020) are predictive of speech understanding performance could clarify the mechanisms underpinning speech-in-noise difficulties in older adults. One potential candidate neural marker is the P300 auditory event-related potential (ERP), which has been shown to be a useful measure for investigating individual differences in cognitive aging (Pinal et al., 2015; Guerrero et al., 2022). The P300 or P3 ERP component is a positive deflection in the electroencephalographic (EEG) waveform that occurs between 300 to 600 ms after stimulus onset, elicited by infrequent task-relevant auditory stimuli interspersed among more frequent standard stimuli. This response is thought to reflect the attentional resources involved in detecting and evaluating unexpected stimuli (Polich, 1992, 2003, 2007). Specifically, the amplitude (magnitude at the peak positive deflection) of the P300 response is thought to be proportional to the amount of attentional resources or energy allocated to the evaluation of the stimulus that evoked the response (Johnson, 1988). The latency of the P300 is thought to reflect the time required to detect and identify the stimulus, independent of processes such as response selection, motor preparation, or execution (Duncan-Johnson, 1981; Verleger, 1997). Evidence from human lesion studies, intracranial recordings, source analysis and fMRI implicate the superior temporal gyrus, parietal–temporal junction and prefrontal cortical regions in the generation of the auditory P300 components (Halgren et al., 1998; Olichney et al., 2022; Soltani and Knight, 2000), regions that are also important for speech perception and attention (Dehaene-Lambertz et al., 2005; Turkeltaub and Coslett, 2010).

Studies using an auditory P300 paradigm where a regular, repeated stimulus is randomly replaced with a deviant, oddball stimulus (Polich et al., 1994; Polich and Heine, 1996) have shown that aging is associated with lower P300 amplitude (van Dinteren et al., 2014) and longer P300 latencies (Brown et al., 1983; Polich et al., 1990; Rossini et al., 2007). The age-related decline in amplitude primarily occurs at parietal electrodes with little-to-no age-related decline at frontal electrodes, due to a “frontal shift” of P300 components in older listeners (Fabiani et al., 1998; Friedman, 2003; Richardson et al., 2011; Alperin et al., 2014; Kamp, 2020). It has been suggested that this shift is due to the recruitment of compensatory frontal processes to evaluate the stimulus (Alperin et al., 2014; van Dinteren et al., 2018). Further, lower amplitudes and longer latencies of the P300 have also been observed in listeners with a variety of neuropsychiatric disorders, including bipolar disorder (Muir et al., 1991; Salisbury et al., 1998; O’Donnell et al., 2004), schizophrenia (Ford, 1999; Jeon and Polich, 2003), Parkinson’s disease (O’Donnell et al., 1987), and dementia (Bonanni et al., 2010; Hedges and Bennett, 2014), relative to unaffected comparison groups. Relatively little work, however, has been conducted on the potential for P300 to capture individual differences in speech understanding in noisy listening environments.

One P300 finding related to speech understanding in noise is that the inclusion of competing background noise in the oddball paradigm results in decreased P300 amplitude and/or increased P300 latency (Polich et al., 1985; Salisbury et al., 2002; McCullagh et al., 2012). Bennett et al. (2012) expanded on this work by considering the relation between P300 responses in noisy conditions and individual differences in speech understanding in noise. They found a significant correlation between P300 peak latency and listener performance in a speech-in-noise identification task that used phonetically balanced IEEE sentences (Egan, 1948) where longer latencies were associated with worse speech recognition performance. However, this result combined EEG measures in three types of background noise (four talker babble, continuous speech-shaped noise, and interrupted speech-shaped noise) and so it is not known how this relationship may differ across the three background noise conditions. In a more recent study, Koerner et al. (2017) used a similar IEEE speech-in-noise recognition task to examine the relation between P300 responses and speech recognition outcomes. While they found no significant correlations between sentence recognition performance and either the amplitude or latency of the P300 response, they did find that inter-trial phase coherence was predictive of sentence recognition. This suggests that consistent neural synchronization (or entrainment) by the speech rhythm of the target speech material is important for speech understanding.

An important common element of these studies to consider is that they use only a single measure of speech identification in noise: keyword identification in spoken low context IEEE sentences. Because speech-in-noise understanding is not a unidimensional construct but rather involves a range of auditory or cognitive processes (e.g., temporal processing and rhythm perception, auditory stream segregation, attention and executive function, and memory), it is likely that different speech-in-noise tasks rely to different degrees on different aspects of speech understanding in noise and moreover P300 responses are not equally predictive of performance for all speech-in-noise measures. This may explain some of the inconsistencies in previous studies investigating the potential relationship between the P300 response and listener’s speech-in-noise comprehension. A more complete picture of the speech-in-noise listening process can be captured by using a wide array of speech-in-noise listening tasks, which should improve our ability to determine if P300 responses are predictive of speech-in-noise understanding.

The aims of the current study were (1) to compare P300 responses in young and older adult listeners in an auditory oddball paradigm involving temporally regular isochronous syllable sequences presented in quiet and noisy (multi-talker babble) background conditions and (2) to investigate the extent to which amplitude and latency of the P300 responses in this oddball paradigm relate to speech-in-noise understanding for a separate battery of speech-in-noise tasks for young and older listeners. Because difficulties in understanding speech in noise are known to be a particularly serious problem for older adults (Plomp and Mimpen, 1979; Dubno et al., 1984), we also considered whether age potentially interacts with the effect of noise on P300 responses by measuring P300 in both quiet and multi-talker babble background conditions. Further, as the amplitude and latency of the P300 are understood to reflect different processes, their correlation with speech-in-noise task performance can provide insight into the mechanisms involved in speech understanding in complex multiple-source environments.

An additional consideration is that stimuli in the oddball paradigm are often presented at regular (equal) temporal intervals (i.e., isochronously), leading to the possibility that listeners anticipate the temporal onset of each successive stimulus and dynamically heighten attention at the expected event onsets. Consistent with this view, rhythmic regularity and temporal expectations have been demonstrated to be important factor in speech understanding (Dilley and McAuley, 2008; Baese-Berk et al., 2019; Shen and Pearson, 2019; McAuley et al., 2020, 2021). In the oddball paradigm, the inclusion of omissions (where a small proportion of stimuli are omitted in the continuous stream of frequent standard and infrequent deviant stimuli) provides a method to investigate correlates of dynamic attending through P300 responses (Jongsma et al., 2005; San Miguel et al., 2013; van Laarhoven et al., 2017; Dercksen et al., 2020). Responses to omissions are thought to represent an unmet expectation, where the amplitude and latency of the response should change based on the strength and accuracy of the temporal expectation listeners have about when the next stimulus will occur (Wacongne et al., 2011; Schröger et al., 2015). Therefore, it is hypothesized that older listeners, who demonstrate age-related deficits in temporal processing and decreased accuracy of timing judgments (Schneider et al., 1994; Fitzgibbons and Gordon-Salant, 1994; Snell and Frisina, 2000; Gordon-Salant and Fitzgibbons, 2004; Lister and Tarver, 2004; Humes et al., 2013), would demonstrate weaker and delayed omission responses compared to young adults.

## 2 Method

### 2.1 Participants

One-hundred and seven native English listeners were recruited from participants who had completed a larger listening test battery. This larger test battery was conducted at two sites, Indiana University—Bloomington and Michigan State University, and all participants at the Indiana University site who met the inclusion criteria were invited to participate in the current study. Listeners were screened for any cognitive, language, or ear-related medical conditions that could affect hearing before participation in the larger test battery. Of the 107 listeners, 35 comprised the older adult group (age criteria = 55 to 90 years, mean age = 68.4 years, age range = 55 to 87 years, 19 female) and 72 comprised the young adult group (mean age = 21.2 years, age range = 18 to 30 years, 44 female). Older adults’ pure-tone average hearing thresholds were calculated at 500, 1,000, and 2,000 Hz; they ranged from 6.67 to 55 dB HL (*M* = 23.13 dB HL) of these older listeners 13 had pure-tone average thresholds >25 dB HL and all but one participant had pure-tone average thresholds >50 dB HL (the outlier had a pure-tone average threshold of 55 dB HL). Pure-tone average hearing thresholds were also collected for young adults who all had hearing thresholds consistent with normal hearing, < 25 dB HL (*M* = 8.36 dB HL) all young adults had normal hearing thresholds through 8,000 Hz.

### 2.2 Stimuli and design

Two naturally produced consonant-vowel (CV) syllables, /ta/ and /ba/, were used to create a continuous isochronous sequence. The CV syllables were recorded by a male talker with a fundamental frequency of approximately 108 Hz and had a 250-ms duration. The sequences were created by continuously presenting 600 CV syllables (either /ta/ or /ba/) with a 200-ms ISI, resulting in a 450 ms inter-onset-interval between syllable onsets corresponding to a presentation rate of slightly faster than 2 Hz. The ~2 Hz rate was selected to be similar to the stress rate of natural speech (Dauer, 1983). One of the syllables was the standard syllable, which was presented 70% of the time. The other syllable was the deviant syllable, which was only presented 15% of the time. For the remaining 15% of sequence elements, the CV syllable was removed creating an omission trial. There were two background conditions: a quiet condition with no background sound and a 6-speaker multi-talker babble condition where 6-speaker multi-talker babble was continuously presented at a +5 dB signal-to-noise ratio throughout the duration of the CV syllable sequence. The syllables were presented at 85 dB SPL in both conditions to ensure audibility for the older listeners.

### 2.3 Procedure

Listeners were seated in a sound attenuated booth and instructed to relax and stay as still as possible throughout the duration of the experiment. Each participant was presented with the continuous sequence of CV syllables binaurally through Etymotic ER-3A insert headphones in two background conditions, one with the sequence presented in quiet and the other with the sequence presented with the 6-talker babble masker. The two background conditions were presented back-to-back with the order of the two conditions randomly assigned for each participant. The oddball paradigm consisted of a 600-element sequence; 70% of the sequence elements were the standard CV syllable, 15% were the deviant syllable, and 15% were omissions where no stimulus was presented. The designation of whether the /ta/ or /ba/ syllable was the deviant syllable was roughly the same across listeners (54 with /ba/ as deviant and 53 with /ta/ as deviant). Listeners were given the identity of the standard stimulus and told to respond only when they heard a deviant syllable.

### 2.4 Data acquisition

Continuous EEG responses were collected from 64 electrode sites using an electrode cap (Falk Minnow Service, Munich, Germany). Data were sampled at 1000 Hz using a Neuroscan SYNAMPS recording system (Neuroscan Inc., El Paso, TX) and impedances were maintained below 10 kΩ. Behavioral responses were made by pressing a button on a button box. The recordings were scheduled after completion of the test battery that contained the speech recognition tasks. The specific time between completion of the speech recognition tasks and the EEG recordings was variable dependent on participant availability.

### 2.5 Data analysis

For the behavioral responses, the percentage of correctly identified deviant syllables (hits) as well as the percentages of false alarm rates for standard and omissions were recorded for each participant. The EEG data were first bandpass filtered from 0.5 to 30 Hz. An independent component analysis was used to separate and remove artifactual activity related to eyeblink activity. EEG data were then separated into three conditions based on the syllable presented (Standard, Deviant, Omission) and then into epochs with a temporal window from 100 ms prior to syllable onset to 700 ms following syllable onset. Automatic artifact rejection was conducted using a threshold of ±100 μV. P300 peak amplitude was measured independently for the Pz, Cz, and Fz electrodes between 300 and 600 ms after syllable onset for both deviant syllables and omissions. Peak amplitude was calculated by first taking the difference between the waveforms for the deviant response and the standard response across all time points and then selecting the peak amplitude of the difference waveform. P300 peak latency was measured by determining the time interval between the syllable onset and the peak amplitude.

Six listeners who did not demonstrate a P300 response to the deviant syllables higher than their response to the standard syllables at any point in the 300–600 ms observation window for all three electrode positions (Pz, Cz, Fz) in both the quiet and multi-talker conditions were removed from further analysis. One more listener was removed for not completing the entire EEG task. This left 101 listeners (71 young, 30 older).

### 2.6 Speech recognition measures

Participants separately completed a battery of speech recognition in noise tasks that was part of a larger listening study. Two standardized speech-in-noise tasks (WIN and QuickSIN) were selected for this study as a representation of listeners’ general ability to understand speech-in-noise. The remaining tasks were based on an earlier test battery (Humes et al., 2013) representing different types of difficult listening conditions. Three Revised Speech-in-Noise (R-SPIN) tasks were selected as a test of listeners’ ability to use context to navigate sub-optimal listening conditions (multi-talker babble background, missing content, and time compressed speech). Finally, two Coordinated Response Measure (CRM) tasks were selected to test listeners’ ability to identify targets at several temporal locations in an utterance while navigating competing speech (1 spoken sentence or 6 talker babble) in a closed set listening task. Tasks from the test battery were administered in a sound attenuated booth through insert earphones (Etymotic ER-3A). The individual tasks were spread across five different sessions. The individual tasks were spread across five different sessions. In general, the sessions were scheduled once or twice a week depending on participant availability and each lasted approximately 2 h. The individual tasks are briefly described below.

#### 2.6.1 Words in noise (WIN)

Listeners were presented with two lists of 35 monosyllabic English words, presented with minimal sentence context (i.e., “Say the word ______”) (Wilson, 2003). The words were presented in four-talker babble at a signal-to-noise ratio (SNR) that started at +24 dB and systematically dropped by 4 dB every five words. Listeners identified each word as they heard it by typing it in a response box. The 50% threshold is estimated from the total number of correct key words using the equation 26—(0.8 × # of correct key words) (Wilson, 2011). The experiment was conducted and scored using a custom script on the E-Prime 3.0 software (Psychology Software Tools, Pittsburgh, PA).

#### 2.6.2 Quick speech-in-noise (Quick-SIN)

12 sentences were presented in the form of two paired lists where each list changed the signal-to-noise ration (SNR) from +25 dB to 0 dB (Killion et al., 2004). Listeners’ task was to identify the words in the sentence and type them into a response box. Listener responses are used to calculate the SNR loss which indicates the general SNR a listener needs to understand speech. The SNR loss is calculated using the equation 25.5—# of keywords correctly identified. The experiment was conducted and scored using a custom script in the E-Prime 3.0 software (Psychology Software Tools, Pittsburgh, PA).

#### 2.6.3 R-SPIN speech recognition tasks

Stimuli for the R-SPIN speech recognition tasks are all taken from the R-SPIN corpus, which consists of simple sentences ending in a monosyllabic noun (Kalikow et al., 1977; Bilger et al., 1984). The materials include 200 predictability-high (PH) sentences in which the final word is highly predictable from the prior context and 200 predictability-low (PL) sentences in which the final word is not predictable from the prior context (i.e., they are presented in a neutral context). In each of the R-SPIN tasks listeners were instructed to listen to each sentence and then type in the last word of each sentence into a response box. The tasks were scored by calculating the overall percent correct word identification. Presentation levels for older listeners were based on their audiograms and were spectrally shaped to provide levels that were at least 13 dB above thresholds for frequencies up to 4,000 Hz. The presentation level for young listeners was set to 85 dB SPL (without shaping) to be comparable to levels used for the older group. The experiments were conducted and scored using a custom script in MATLAB software (The MathWorks Inc., 2024).

Babble SPIN (BSPIN): The SPIN task was performed with sentences presented in the original R-SPIN 12-talker babble at +8 dB SNR (following Humes et al., 2013). The task consisted of 100 PH sentences and 100 PL sentences. Data is presented separately for the two predictability conditions.

Interrupted SPIN (ISPIN): In this task, the SPIN sentences were altered by cutting out portions of the audio and replacing them with an equal period of silence. This simulates a difficult listening situation in which portions of the signal are inaudible and listeners must use limited information to identify the final word in the sentence. An isochronous pattern of “glimpses” of the speech was used throughout the sentence, with the glimpse duration based on the target-word duration, such that all target words included eight equal-duration glimpses comprising 50% of the total word duration (Wang and Humes, 2010). These stimuli were presented in speech-shaped noise at a +10 dB SNR. The task consisted of a different set of 100 PH sentences and 100 PL sentences than used in the Babble Spin task. Data is presented separately for the two predictability conditions.

Time Compressed SPIN (TCSPIN): For this task, a random selection of 100 PL SPIN sentences were time compressed using a 50% ratio, resulting in halved sentence durations. Time compression was achieved using a uniform compression algorithm (Gordon-Salant and Fitzgibbons, 1993).

#### 2.6.4 Coordinated response measure (CRM) tasks

All of the speech materials in the CRM corpus (Bolia et al., 2000) follow a consistent template: “Ready [call sign] go to [color] [number] now.” The CRM sentence set includes eight call signs (arrow, baron, charlie, eagle, hopper, laker, ringo, tiger), four colors (blue, green, red, white), and eight numbers (1 through 8). Listeners’ task was to identify the color and number of the target sentence in each trial. The target sentence always used the call sign “Baron” while the color and number were randomly selected. The talker for the target sentence consisted of either a male or female talker. Two versions of this listening task were used in this study. The first had a competing background consisting of another CRM sentence with a different call sign, color, and number spoken by a different-sex talker (CRM1). The second version replaced the competing CRM sentence with six talker babble consisting of three male voices and three female voices all using different call signs, colors, and numbers than the target sentence. The single competitor and six talker babble conditions create different masking issues for the listener. Six-talker babble creates a steadier masker that could potentially create more energetic masking (e.g., moving from two talker to six talker babble degrades performance) (Humes et al., 2017), but the similarity of a single talker producing a similar sentence can also create additional informational masking that would be expected to lead to more difficulty than the six-talker babble (Humes et al., 2013). Prior to the start of these two tasks, listeners practiced with a version of this task without background sounds, to familiarize themselves with the process. For the older listener group, spectral shaping was applied to each 1/3 octave band to produce speech 13 dB above the listener hearing threshold. Young listeners were presented with a presentation level of 85 dB SPL in order to be similar to the presentation level used for older listeners. The signal-to-noise ratio was set to −1 dB for both age groups. For each condition there were four blocks consisting of 32 sentences. Percentage of correct responses (correctly reporting both color and number correct) were recorded. The CRM tasks were administered and scored using custom scripts in MATLAB.

## 3 Results

### 3.1 EEG results

EEG recordings were obtained while participants performed the oddball task described above, in which they were required to identify deviant syllables by pressing a button. The 101 participants demonstrated highly accurate identification of the deviant syllable with a 97.4% hit rate. False alarm rates were also very low. Out of the 420 standard syllables in each background condition, listeners on average incorrectly identified only 0.5% of the standards as a deviant syllable. Out of the 90 omissions, only 0.7% were incorrectly identified as a deviant syllable. Overall, the behavioral results show near ceiling performance in both young adults (deviant HR = 97%, standard FAR = 0.4%, omission FAR = 0.7%) and older adults (deviant HR = 98.2%, standard FAR = 0.6%, omission FAR = 0.7%). Similarly, there are no differences between the quiet (deviant HR = 97.7%, standard FAR = 0.4%, omission FAR = 0.7%) and multi-talker background conditions (deviant HR = 97.1%, standard FAR = 0.5%, omission FAR = 0.7%) pooled across age groups.

### 3.2 Deviant syllables

**Figure 1:**
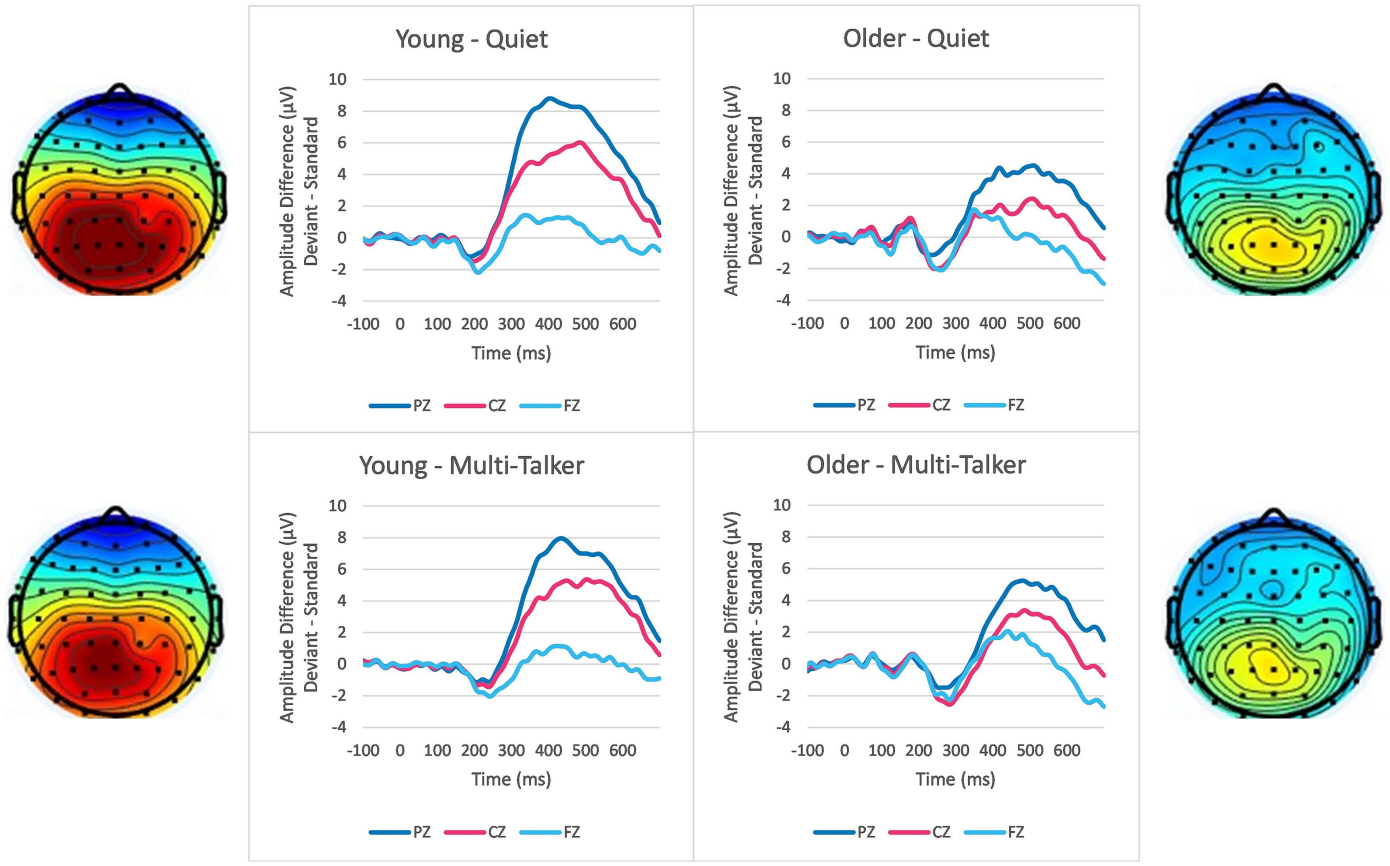
Grand average difference waveforms showing the difference between deviant syllable and standard syllable responses separated by age group (left column: young; right column: older), background condition (top row: quiet; bottom row: multi-talker babble) and the three electrode positions (different lines). Scalp topography depicting mean P300 amplitude between the latencies of 300–600 ms are shown for young listeners (left side) and older listeners (right side) in quiet (top) and multi-talker (bottom) conditions. The scale for the scalp topography ranges from −2 μVs to 6 μVs.

Mean P300 peak amplitudes and peak latencies in response to deviant syllables at electrodes Pz, Cz, and Fz in the quiet and multi-talker babble conditions are shown in Tables 1, 2. The corresponding grand average difference waveforms are shown in Figure 1. P300 amplitudes and latencies values were in the expected ranges based on previous P300 studies (Salisbury et al., 2002; Koerner et al., 2017).

**Table 1:** Mean and standard deviation of P300 amplitude values (measured as peak difference between deviant and standard syllable response amplitudes) for the two background conditions (quiet and multi-talker babble), three electrode locations, and two age groups (young: *n* = 71, older: *n* = 30).

P300 peak amplitude (μv)						
Age	Quiet			Multi-talker babble		
	Pz***	Cz***	Fz	Pz*	Cz*	Fz
Young	10.40 (4.68)	7.99 (4.30)	3.92 (3.10)	9.02 (4.75)	6.99 (4.67)	3.61 (3.17)
Older	6.68 (3.59)	4.41 (4.93)	3.40 (4.30)	6.92 (3.87)	4.87 (4.39)	3.53 (3.80)

Significant differences in P300 amplitude comparing young and older adults at each of the electrode locations are indicated with *, ** and *** representing *p* < 0.05, *p* < 0.01 and *p* < 0.001 levels, respectively.

**Table 2:** Mean and standard deviation of peak latency values for P300 responses to deviant stimuli for the two background conditions (quiet and multi-talker babble), three electrode locations, and two age groups (young: *n* = 71, older: *n* = 30).

P300 peak latency (ms)						
Age	Quiet			Multi-talker babble		
	Pz***	Cz**	Fz	Pz***	Cz**	Fz
Young	385 (46.05)	383 (57.07)	381 (66.39)	405 (44.99)	413 (60.67)	421 (66.42)
Older	424 (62.02)	422 (70.20)	401 (68.02)	454 (65.85)	452 (69.24)	434 (65.75)

Significant differences in P300 amplitude comparing young and older adults at each of the electrode locations are indicated with *, ** and *** representing *p* < 0.05, *p* < 0.01 and *p* < 0.001 levels, respectively.

To investigate the effects of age, background condition, and electrode location on P300 responses to a deviant syllable, separate 2 (age group: young, older) × 2 (background condition: quiet, multi-talker babble) × 3 (electrode location: Pz, Cz, Fz) mixed-measures ANOVA were conducted on P300 peak amplitude and peak latency values separately. For amplitude, there were significant main effects of age group, *F* (1,99) = 7.354, *p* = 0.008, *η*^2^ = 0.069, and electrode location, *F* (2,198) = 119.596, *p* = < 0.001, *η*^2^ = 0.547, showing higher P300 peak amplitudes for young listeners compared to older listeners and for more parietal electrode locations. There was no significant main effect of background condition, *F* (1,99) = 0.757, *p* = 0.386, *η*^2^ = 0.008. There was also a significant interaction between age group and electrode location, *F* (2,198) = 12.296, *p* = < 0.001, *η*^2^ = 0.110. There were no other significant interactions (all *p*’s > 0.05). Overall, P300 peak amplitudes were significantly lower for older adults, M = 4.98, SD = 4.55, compared to young adults, M = 6.991, SD = 4.89 although the effect size was smaller than that seen for electrode location. With respect to electrode location, post-hoc comparisons applying a Bonferroni correction showed that the mean amplitude of the responses at Pz was significantly higher than at both Cz, M = 2. 189, *p* < 0.001, and Fz, M = 4.641, *p* < 0.001, and that amplitudes at Cz were also significantly higher than at Fz, M = 2.452, *p* < 0.001. To investigate the interaction between age group and electrode location, independent-sample t-tests were conducted on P300 peak amplitudes averaged across the two background conditions. The results found that while young listeners had significantly greater P300 amplitude than older listeners at Pz, t (99) = 3.857, *p* = 0.001, and Cz, t (99) = 3.622, *p* < 0.001, there was no significant difference between the two age groups at Fz, t (99) = 0.684, *p* = 0.248.

With respect to P300 peak latency values, the ANOVA revealed significant main effects of age group, *F* (1,99) = 11.230, *p* = 0.001, *η*^2^ = 0.102, background condition, *F* (1,99) = 18.413, *p* < 0.001, *η*^2^ = 0.157, and electrode location, *F* (2,198) = 3.224, *p* = 0.042, *η*^2^ = 0.032. There was also a significant interaction between age group and electrode location, *F* (2,198) = 3.24, *p* < 0.001, *η*^2^ = 0.076. There were no other significant interactions (all *p*’s > 0.05). Overall, P300 peak latencies were longer for older adults, M = 431.21, SD = 70.08, than for young adults, M = 398.08, SD = 60.51. Peak latencies were also longer for the multi-talker babble background, M = 429.88, SD = 62.19, than for the quiet background, M = 399.40, SD = 66.47. For the main effect of electrode location, post-hoc comparisons applying a Bonferroni correction only showed a significant difference between the responses at Cz and Fz, with Fz having shorter latencies than Cz, M = −8.040, *p* = 0.047. A similar pattern was found for Fz having shorter latencies than Pz, but this was not statistically significant, M_diff_ = −7.786, *p* = 0.258, while there was almost no difference between the mean latencies at Pz and Cz, M_diff_ = −0.254, *p* = 0.999. To investigate the interaction between electrode location and age group, independent sample t-tests were conducted on P300 peak latencies averaged across the quiet and multi-talker babble backgrounds. Similar to the peak amplitude results, peak latencies for young listeners were significantly shorter than older listeners at Pz, t (99) = −3.433, *p* < 0.001, and Cz, t (99) = −2.847, *p* = 0.003, but not significantly different at Fz, t (99) = −1.363, *p* = 0.088.

### 3.3 Omissions

Mean P300 peak amplitudes and latencies in response to omissions at electrodes Pz, Cz, and Fz are shown in Tables 3, 4. A 2 (age group: young, older) × 2 (background condition: quiet, multi-talker babble) × 3 (electrode location: Pz, Cz, Fz) mixed-measures ANOVAs on amplitude values revealed a main effect of age group, *F* (1,99) = 7.408, *p* = 0.008, *η*^2^ = 0.008, and electrode location, *F* (1,99) = 65.314, *p* < 0.001, *η*^2^ = 0.397, demonstrating higher peak amplitudes in the young listener group and for more frontal electrodes. There was no significant main effect of background condition, *F* (1,99) = 1.341, *p* = 0.250, *η*^2^ = 0.013. There were also no significant interactions (all *p*’s > 0.05). Bonferroni-corrected post-hoc comparisons examining the main effect of electrode location showed that mean P300 peak amplitudes were significantly higher at Fz than at both Cz, M_diff_ = 0.510, *p* = 0.003, and Pz, M_diff_ = 1.941, *p* < 0.001, electrodes while the responses at Cz were also significantly higher than at Pz, M_diff_ = 1.431, *p* < 0.001.

**Table 3:** Mean and standard deviation of P300 amplitude values (measured as peak difference between omission and standard syllable response amplitudes) for the two background conditions (quiet and multi-talker babble), three electrode locations, and two age groups (young: *n* = 71, older: *n* = 30).

Omissions P300 peak amplitude (μv)						
Age	Quiet			Multi-talker babble		
	Pz	Cz**	Fz**	Pz**	Cz*	Fz
Young	3.73 (2.78)	5.61 (2.93)	6.03 (2.92)	3.61 (2.50)	4.81 (2.21)	5.30 (2.26)
Older	2.85 (1.92)	4.01 (2.35)	4.57 (2.51)	2.44 (2.18)	3.92 (2.83)	4.49 (3.86)

Significant differences in P300 amplitude comparing young and older adults at each of the electrode locations are indicated with *, ** and *** representing *p* = 0.05, *p* = 0.01 and *p* = 0.001 levels, respectively.

**Table 4:** Mean and standard deviation of peak latency values for P300 responses to trials where the stimuli was omitted for the two background conditions (quiet and multi-talker babble), three electrode locations, and two age groups (young: *n* = 71, older: *n* = 30).

Omissions P300 peak latency (ms)						
Age	Quiet			Multi-talker babble		
	Pz*	Cz*	Fz***	Pz	Cz	Fz*
Young	383 (65.16)	379 (63.70)	367 (55.18)	391 (67.39)	379 (56.52)	369 (49.20)
Older	411 (74.60)	407 (71.23)	409 (66.43)	390 (61.79)	400 (73.81)	391 (67.51)

Significant differences in P300 amplitude comparing young and older adults at each of the electrode locations are indicated with *, ** and *** representing *p* = 0.05, *p* = 0.01 and *p* = 0.001 levels, respectively.

For latency values, the corresponding ANOVA revealed significant main effects for age group, *F* (1,99) = 5.625, *p* = 0.020, *η*^2^ = 0.054, and location, *F* (2,198) = 3.337, *p* = 0.038, *η*^2^ = 0.033, with shorter latencies for young listeners than for older listeners and at more frontal electrodes than more parietal electrodes. To further investigate the location effect a paired samples t test was conducted to compare latencies across electrodes. The analysis found that in both the quiet and multi-talker conditions there was no significant difference between Pz and Cz (Quiet: t (100) = 1.062, *p* = 0.291; Multi-talker: t (100) = 1.044, *p* = 0.299), while the Fz electrode had significantly shorter latencies than the Pz (Quiet: t (100) = 2.513, *p* = 0.014; Multi-talker: t (100) = 2.634, *p* = 0.010) and Cz (Quiet: t (100) = 2.139, *p* = 0.035; Multi-talker: t (100) = 2.326, *p* = 0.022). There was no significant main effect of background condition, *F* (1,99) = 0.598, *p* = 0.441, *η*^2^ = 0.006, and there were also no significant interactions (all *p* > 0.05).

### 3.4 Correlations between speech-in-noise performance and deviant P300 amplitude and latency

Of the 101 listeners in the current study, 3 of them (1 older listener) did not complete the entire battery of speech-in-noise tests that was part of a larger study. These listeners were removed from the correlational analysis conducted on the pooled data from both the young and older group reported below. In general, the young adults outperformed the older adults on five of the nine speech-in-noise tasks (WIN, QSIN, TCSPIN, CRM1, and CRM6); however, older adults outperformed the young adults on the ISPIN LP and ISPIN HP tasks. The better ISPIN performance for older listeners is consistent with similar results by Humes et al. (2013). It may be that, at least for this type of missing information (interrupted words) in a sentence context, older listeners’ greater experience with hearing loss may have led to better use of context and a better ability to fill in missing information. For the remaining two tasks (BSPIN_LP and BSPIN_HP) there were no significant differences between young and older listeners (see Table 5).

**Table 5:** Mean performance (proportion correct (PC) or signal-to-noise ratio (SNR)) with standard deviations in parentheses for the nine speech-in-noise measures for the young adults (*n* = 69) and older adults (*n* = 29).

SIN measure	Young (*n* = 69)	Older (*n* = 29)
WIN*** (50% correct SNR)	5.51 dB (1.23)	8.78 dB (2.96)
QSIN*** (SNR Loss)	2.96 dB (1.43)	4.665 dB (2.57)
BSPIN_LP (PC)	0.68 (0.11)	0.68 (0.14)
BSPIN_HP (PC)	0.96 (0.12)	0.98 (0.03)
ISPIN_LP*** (PC)	0.35 (0.11)	0.50 (0.11)
ISPIN_HP*** (PC)	0.60 (0.16)	0.86 (0.13)
TCSPIN*** (PC)	0.89 (0.12)	0.79 (0.15)
CRM1M** (PC)	0.48 (0.14)	0.33 (0.08)
CRM6M** (PC)	0.62 (0.16)	0.49 (0.015)

Significant differences between young and older adults indicated with *, ** and *** representing *p* = 0.05, *p* = 0.01 and *p* = 0.001 levels, respectively.

P300 peak amplitude values in response to deviant stimuli were correlated with scores on the nine speech-in-noise tasks in quiet and multi-talker babble background conditions (see Table 6). The same correlational analysis was then conducted using P300 peak latency values. For P300 amplitude in the quiet condition, significant correlations were found with WIN (Pz: (r(96) = 0.318, *p* = 0.001), Cz: (r(96) = 0.224, *p* = 0.026), Fz: (r(96) = 0.224, *p* = 0.027)), ISPIN LP (Pz: (r(96) = 0.200, *p* = 0.05)), and ISPIN HP (Pz: (r(96) = 0.255, *p* = 0.012)). For P300 amplitude in the multi-talker babble condition, significant correlations were found with WIN (Pz: r(96) = 0.384, *p* < 0.001, Cz: r(96) = 0.269, *p* = 0.007), QSIN (Pz: r(96) = 0.281, *p* = 0.005), ISPIN LP (Pz: (r(96) = 0.298, *p* = 0.003), Cz: (r(96) = 0.207, *p* = 0.045)), and ISPIN HP (Pz: r(96) = 0.286, *p* = 0.005)). None of the speech-in-noise measures were found to reliably correlate with latency (all *p*’s > 0.05).

**Table 6:** Pearson correlations between P300 peak amplitudes at Pz, Cz, and Fz (in both quiet and multi-talker babble) with performance on the nine speech-in-noise measures is presented for all listeners (*n* = 99).

	Quiet—Pz	Quiet—Cz	Quiet—Fz
WIN	r(96) = −0.326, *p* = 0.001***	r(96) = −0.324, *p* = 0.001***	r(96) = −0.150, *p* = 0.139
QSIN	r(96) = −0.089, *p* = 0.382	r(96) = −0.099, *p* = 0.333	r(96) = −0.009, *p* = 0.933
BSPIN LP	r(96) =0.024, *p* = 0.813	r(96) = 0.037, *p* = 0.721	r(96) = −0.057, *p* = 0.579
BSPIN HP	r(96) = −0.079, *p* = 0.441	r(96) = −0.099, *p* = 0.331	r(96) = −0.105, *p* = 0.301
ISPIN LP	r(96) = −0.238, *p* = 0.019*	r(96) = −0.156, *p* = 0.127	r(96) = −0.012, *p* = 0.908
ISPIN HP	r(96) = −0.276, *p* = 0.006**	r(96) = −0.245, *p* = 0.015*	r(96) = −0.072, *p* = 0.484
TCSPIN	r(96) = 0.120, *p* = 0.240	r(96) = 0.068, *p* = 0.510	r(6) = −0.060, *p* = 0.556
CRM1	r(968) = 0.207, *p* = 0.042*	r(96) = 0.174, *p* = 0.088	r(96) = −0.075, *p* = 0.466
CRM6	r(96) = 0.093, *p* = 0.363	r(96) = −0.054, *p* = 0.598	r(96) = −0.163, *p* = 0.111

	Multi-talker—Pz	Multi-talker—Cz	Multi-talker—Fz
WIN	r(96) = −0.248, *p* = 0.014*	r(96) = −0.285, *p* = 0.003**	r(96) = −0.219, *p* = 0.030*
QSIN	r(96) = −0.115, *p* = 0.260	r(96) = −0.195, *p* = 0.055	r(96) = −0.173, *p* = 0.089
BSPIN LP	r(96) = 0.195, *p* = 0.055	r(96) = 0.192, *p* = 0.058	r(96) = 0.184, *p* = 0.070
BSPIN HP	r(96) = 0.002, *p* = 0.847	r(96) = 0.053, *p* = 0.607	r(96) = 0.180, *p* = 0.076
ISPIN LP	r(96) = −0.025, *p* = 0.806	r(96) = −0.029, *p* = 0.781	r(96) = −0.093, *p* = 0.363
ISPIN HP	r(96) = −0.037, *p* = 0.720	r(96) = −0.052, *p* = 0.614	r(96) = 0.127, *p* = 0.213
TCSPIN	r(96) = 0.152, *p* = 0.137	r(96) = 0.169, *p* = 0.098	r(96) = 0.182, *p* = 0.075
CRM1	r(96) = 0.194, *p* = 0.062	r(96) = 0.201, *p* = 0.048*	r(96) = 0.149, *p* = 0.145
CRM6	r(96) = 0.169, *p* = 0.098	r(96) = 0.133, *p* = 0.195	r(96) = 0.139, *p* = 0.175

Significant differences between young and older adults indicated with *, ** and *** representing *p* = 0.05, *p* = 0.01 and *p* = 0.001 levels, respectively.

**Figure 2:**
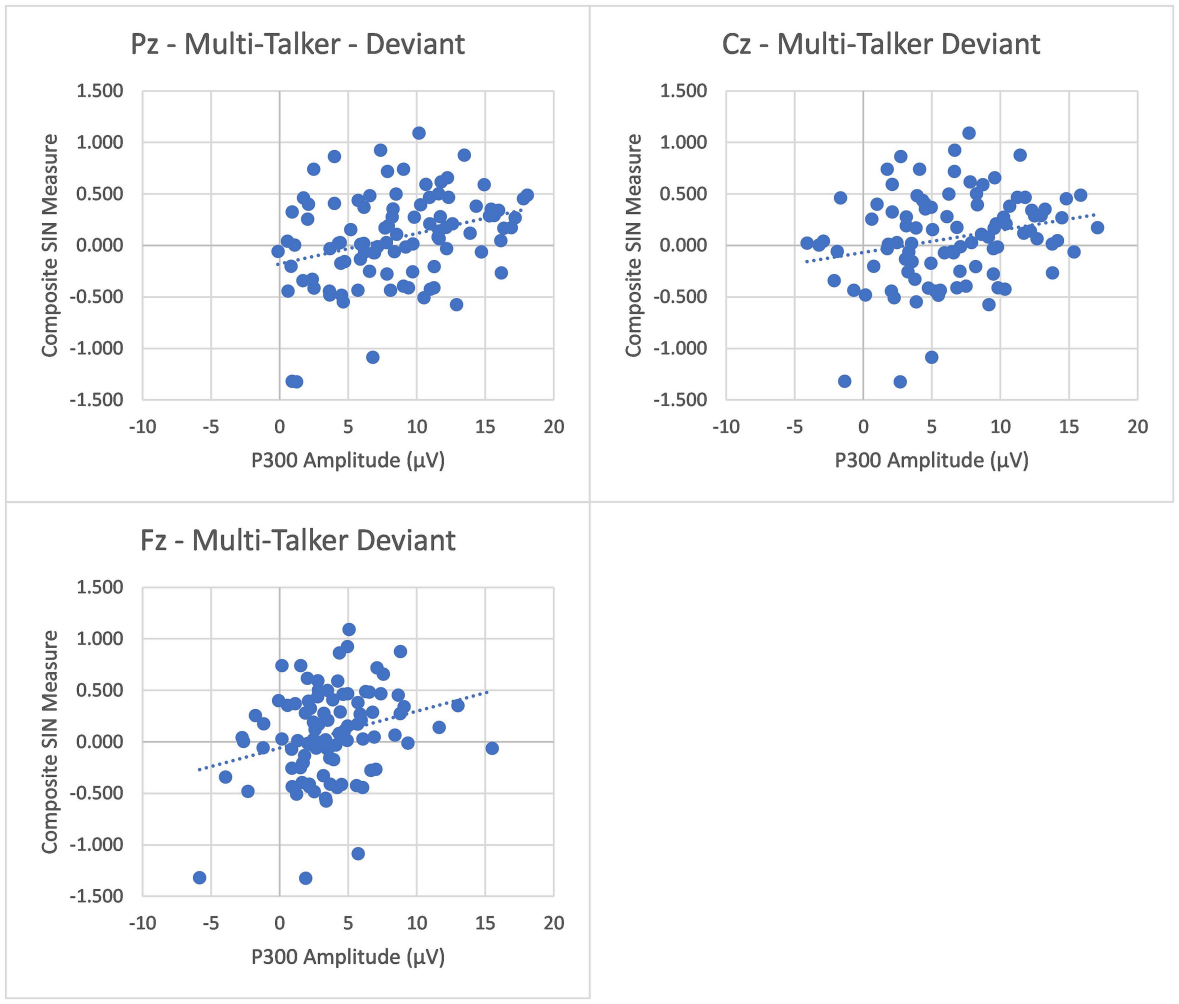
Pearson correlations between the Composite SIN measures and the P300 peak amplitudes at Pz (Top-Left Panel), Cz (Top-Right Panel), and Fz (Bottom-Left Panel) in both multi-talker babble are presented. The peak amplitude at all three electrodes demonstrate a significant positive correlation with the Composite SIN measure.

To further investigate the relationship between P300 and speech-in-noise understanding, we examined the relationship between P300 peak amplitude and latency measures and a composite speech-in-noise measure that combined scores for all nine speech-in-noise measures. To construct the composite measure, scores for each of the 9 speech-in-noise tasks were first z-transformed and then averaged (with all measures scaled such that higher scores indicated better performance). The composite measures for two listeners (1 older adult) were greater than 2 standard deviations from the mean and were removed prior to analysis. There were significant positive correlations between P300 amplitude in the multi-talker condition and the composite measure at all three electrodes (Pz: (r(94) = 0.316, *p* = 0.002), Cz: (r(94) = 0.233, *p* = 0.023), Fz: (r(94) = 0.274, *p* = 0.007)), while no significant correlations were found between P300 amplitude in the quiet condition at any electrode (Pz: (r(94) = 0.096, *p* = 0.350), Cz: (r(94) = 0.062, *p* = 0.546), Fz: (r(94) = −0.045, *p* = 0.661)). Plots for the multi-talker condition correlation can be seen in Figure 2. In order to ensure that this correlation was not just an artifact of age differences in the composite SIN measure and P300 amplitude, a second correlational analysis was conducted with only the young listeners. The analysis again found significant positive correlation between P300 amplitude in the multi-talker condition at all three electrodes (Pz: (r(68) = 0.346, *p* = 0.004), Cz: (r(68) = 0.273, *p* = 0.024), Fz: (r(68) = 0.401, *p <* 0.001)), but no significant correlations were found at any electrode in the quiet condition (Pz: (r(68) = 0.064, *p* = 0.605), Cz: (r(68) = 0.07, *p* = 0.572), Fz: (r(68) = 0.003, *p* = 0.979)). These correlations demonstrate an association between greater P300 peak amplitudes and better speech-in-noise listening performance. No significant correlations were found between P300 peak latency and the composite measure of speech in noise for any electrode in either the quiet or multi-talker conditions (all *p*’s > 0.05).

### 3.5 Predicting speech-in-noise performance using omission P300 amplitude and latency

The previous correlational analysis was repeated for the P300 peak amplitude and latency values in response to the omission trials. In terms of the composite speech-in-noise measure, no significant correlation was found for the peak amplitude or the latency of the omission response measure in either quiet or the multi-talker background (all *p*’s > 0.05). Correlation with performance in the individual speech-in-noise tasks reveals a significant correlation between the QSIN performance and omission amplitude in multi-talker background at Fz (r(96) = −0.201 *p* = 0.047) indicating that better performance in the QSIN task is associated with greater P300 omission amplitude. Further, there was a negative correlation with the ISPIN_HP, at Pz only, similar to the negative correlation seen in the deviant trials (r(96) = −0.244, *p* = 0.016). The omission amplitude in the quiet condition showed significant correlation with the single talker background condition of the CRM task at Pz (r(96) = 0.216, *p* = 0.034) and Cz (r(96) = 0.220, *p* = 0.03). These findings suggest that while there was no correlation between the composite speech-in-noise measure, response amplitude elicited by omission trials may be connected to listener performance in speech-in-noise conditions. No significant correlations were found for the multi-talker condition response latencies. For the P300 latencies measured in the quiet background condition, the primary finding was the correlation between ISPIN_LP (Pz: r(96) = 0.287, *p* = 0.004; Cz: r(96) = 0.235, *p* = 0.02; Fz: r(96) = 0.220, *p* = 0.03) and ISPIN_HP (Pz: r(96) = 0.252, *p* = 0.013; Cz: r(96) = 0.216, *p* = 0.034; Fz: r(98) = 0.244, *p* = 0.016). Similar to the previous ISPIN correlations these show a pattern of better ISPIN performance leading to longer latency in the listener omission response. There was an additional significant correlation between latency and the BSPIN_LP at Pz only (r(96) = 0.235, *p* = 0.02), suggesting an association between response latencies to omission trials and speech comprehension in noise.

## 4 Discussion

The current study was conducted in order to (1) compare peak P300 amplitude and latencies in young and older listeners elicited by deviant syllables and omissions in an oddball detection task presented in quiet and multi-talker babble background conditions and (2) investigate potential associations between P300 responses and speech-in-noise understanding in young and older adults. To this end, an oddball paradigm including deviant syllables, standard syllables, and omissions was used to elicit P300 responses from young and older listeners in both quiet and multi-talker background conditions. Peak amplitudes and latencies of deviant P300 responses were then correlated with listener performance on a wide range of speech recognition tasks (administered in a separate session) which have been previously used to measure listeners’ ability to understand speech in noisy or multi-talker environments.

In terms of P300 and aging, there were three main findings. Analysis of P300 responses found that older listeners had lower amplitudes and longer latencies, relative to young listeners, in response to the deviant syllable. Older listeners also had a more frontal distribution of activity with their P300 response amplitude at Fz being stronger relative to the Pz response when compared to young listeners. While older listeners had lower amplitudes and longer latencies at Pz and Cz, their Fz responses were not significantly different than those of young listeners. Finally, in response to omissions, older listeners demonstrated lower amplitudes and longer latencies relative to young listeners. With respect to the question of the potential for P300 responses to relate to speech-in-noise understanding, the correlational analysis revealed a small but significant correlation between P300 amplitude in deviant trials and a composite measure of speech in noise (which combined performance across the nine speech recognition tasks), but only for P300 amplitudes measured in multi-talker babble.

### 4.1 Effects of age on P300

Consistent with previous studies (Brown et al., 1983; Polich et al., 1990; Rossini et al., 2007; McCullagh and Shinn, 2018), older listeners demonstrated lower P300 peak amplitudes and longer peak latencies for both quiet and multi-talker background conditions. The observed age differences may reflect reduced attentional resources available to older adults (Craik, 1982) as well as a reduced ability to accurately anticipate the onset of events in the isochronous oddball task. A listener having lower P300 amplitude in response to a deviant event may be allocating less attentional energy at the onset of the deviant stimulus (Johnson, 1988) and may also have a smaller pool of attentional resources available for allocation (Kahneman and Tversky, 1973). This finding suggests that lower P300 amplitudes may represent degraded dynamic attending processes in older listeners. Age differences in P300 amplitude may also be due to older adults having to expend greater effort to perform the task, especially when multi-talker babble is present. When listeners are asked to perform an additional task while measuring P300 responses in an oddball paradigm, amplitude decreases as the additional task is more difficult and attention-demanding (Kok, 2001; Polich, 1996) and this effect of task difficulty is generally greater for older and hearing-impaired listeners (Bertoli and Bodmer, 2014).

As for P300 latency, longer latencies are found to be correlated with a decrease in processing speed (Polich et al., 1983; Johnson et al., 1985; Emmerson et al., 1989). Slowed processing speed has been associated with aging (Salthouse and Ferrer-Caja, 2003) which suggests that age-related differences in P300 latency observed in the present study may result from older listeners needing more time to evaluate the deviant stimulus. The prolonged latency is also consistent with less accuracy in anticipating the onset of events in the isochronous oddball task. Thus, both the lower amplitude and longer latency observed with older listeners may be due, at least in part, to poorer entrainment and less accurate anticipation of events in the current task.

There was also a significant interaction between age group and the location of the electrodes. The topographic data in Figure 1 show how P300 amplitude is spatially distributed across electrode locations for the two age groups. Young listeners demonstrate a consistent, strong parietal response to the deviant syllable presentation. This is an expected response to a deviant event, like the deviant syllable in the current study (Ruchkin et al., 1987). In comparison to young listeners, the difference between the parietal response of older listeners and the response at electrodes at Cz and Fz was reduced. While they similarly demonstrated the strongest responses at the parietal electrodes, they also had a more frontal response overall than the young listener group, resulting in a response more equally distributed than the response of young listeners. This increase in frontal activity fits in the with the “frontal shift” commonly found for older listeners (Fabiani et al., 1998; Friedman, 2003; Richardson et al., 2011; Alperin et al., 2014; Kamp, 2020). The most common interpretation of the frontal shift is that older listeners find the task more difficult and must compensate by recruiting additional cognitive processes from frontal regions, potentially in the prefrontal cortex (van Dinteren et al., 2018). Combined with lower amplitudes across electrodes, this explanation reinforces the idea that older listeners’ rhythm-based expectations are weaker than those of younger listeners, resulting in less attentional energy allocated at expected event onsets and more reliance on post-event evaluation of the stimulus. Another potential explanation for the frontal shift is that older listeners demonstrate less habituation to a repeated stimulus (Friedman et al., 1998; O’Connell et al., 2012), although more recent studies have found little evidence to support this explanation (Alperin et al., 2014; Kamp, 2020). Reduced habituation to the standard stimulus would reduce the novelty of the deviant stimulus, which has been shown to reduce P300 amplitude (Gonsalvez and Polich, 2002). These explanations are not exclusive of each other and both explanations suggest that attentional allocation and expectancies are important factors in age-related speech understanding deficits.

Older listeners also demonstrated significantly lower amplitudes and longer latencies in P300 responses to syllable omissions. Omission responses occur due to an unexpected silence created through the consistent regular stimulus timing used in the oddball paradigm. Within this framework, an omission response is considered to reflect a response to an unmet auditory expectation (Wacongne et al., 2011; Schröger et al., 2015) devoid of any sensory processing (Arnal and Giraud, 2012). Specifically, the P300 is the highest latency component to omission responses and is believed to reflect attention and expectation updating (Polich, 2007; Baldi and Itti, 2010). From this, the lower amplitude and longer latency in older listeners may reflect weaker or less precise temporal expectations which delay their identification of an omission further from the regular vowel onset time. This potentially reflects age-related differences in the response to regular, predictable timing. From a dynamic attending theory framework, temporal expectations are the result of the entrainment of internal attentional rhythms by an external rhythmic structure (Jones and Boltz, 1989; Large and Jones, 1999). Poorer entrainment in older adults may lead to weaker and less accurate temporal expectations which may explain the attentional deficits underlying the age-related differences in P300 responses in the current study. Due to the highly regular temporal structure of the oddball paradigm employed here, this suggests a potential connection between individual differences in listener entrainment and the P300 responses elicited from these tasks.

### 4.2 P300 amplitude as an index of speech-in-noise understanding

Previous studies have been inconsistent in demonstrating reliable correlations between P300 responses and specific speech-in-noise tests (Bennett et al., 2012; Koerner et al., 2017). The current study further explored this connection by examining correlations between P300 and multiple speech recognition tasks. Listening to and understanding speech in complex, multiple-source environments is a challenging and complex task that is unlikely to involve a single auditory ability, and different listening tasks may involve different listening abilities to different degrees. Therefore, in order to provide a measure of speech-in-noise understanding ability that captures the abilities utilized across all of the different speech-in-noise tasks, a composite measure of speech in noise was created for each individual based on their performance across all nine speech-recognition tasks. Significant positive correlations between this composite measure and the P300 responses were found in the multi-talker babble condition across all three electrode locations, but no significant correlations were found in the quiet condition. These correlations were also demonstrated in the young listening group independently of the older listeners. It is important to note that although a subset of the older listeners had some degree of hearing loss (13 participants) it has been demonstrated that the stimulus intensity does not have a significant effect on P300 amplitude (Papanicolaou et al., 1985), which suggests that hearing loss was not a factor in these correlations. The finding of a relation between P300 amplitude and speech-in-noise understanding only for evoked responses in the multi-talker condition indicates that the P300 is a useful measure of the attentional demands of speech-in-noise listening only when the evoked responses are collected under difficult listening conditions. For P300 latency, no significant correlation was found in either background condition. These findings demonstrate that the amplitude of the P300 response can function as a neural predictor of listener speech-in-noise comprehension. Previous inconsistency demonstrating this correlation may be due to the difficulty of capturing a listener’s ability to listen in complex environments using a single task.

P300 amplitude is believed to reflect the amount of attentional energy directed to a target (Johnson, 1988). From this, P300 amplitude can be seen as a measure of dynamic attending as listeners attention is directed across the isochronous CV syllable presented in the experiment. The correlation between P300 amplitude and the composite measure of speech in noise highlights the role of dynamic attending in successful navigation of complex, multiple-source auditory environments. This fits with established research into selective entrainment which has demonstrated that listeners are more successful understanding speech in complex environments that facilitate attending to the target signal (i.e., where the target has a regular rhythmic structure) (McAuley et al., 2020).

Looking into individual speech task correlations, the ISPIN task (high and low predictability) stands out in that it showed a negative correlation with peak P300 amplitude and a positive correlation with P300 latency at the Pz electrode (where P300 responses were strongest). The ISPIN task was the only task in the speech recognition battery where the older listener group outperformed the young listener group. This age advantage, combined with the negative correlation, suggests that the filling-in of missing information, based on context, required by the ISPIN task involves a cognitive ability that is independent of entrainment to the speech stimuli. This idea is reinforced by the significant positive correlation found between ISPIN task performance and P300 latency. As the P300 latency is thought to reflect the time required to detect and identify the presented stimulus (Duncan-Johnson, 1981; Verleger, 1997), rather than attention allocation, this correlation fits with the assumption that ISPIN performance is less dependent upon dynamic attending. The latency of the P300 evoked in multi-talker conditions correlated with two other speech-in-noise tasks: the WIN and the Quick-SIN. These tasks involve the identification of speech (both isolated words and sentences) in a noisy background which is similar to the IEEE sentence-identification task used in Bennett et al. (2012), which found a correlation between latency and speech-in-noise understanding. This correlation supports the previous finding suggesting a relationship between P300 latency and some components of speech-in-noise listening, despite the current study not finding a correlation between P300 latency and the composite measure of listeners ability to understand speech in noise.

## 5 Conclusion

The current study used an auditory oddball paradigm involving the presentation of frequent (standard) syllables and infrequent (deviant) syllables in an isochronous sequence to (1) examine P300 responses in quiet and multi-talker background conditions in young and older listeners and (2) assess the use of P300 as an index of speech understanding in noise. Older listeners’ P300 responses to deviant syllables were found to have lower amplitude and longer latency, as well as having relatively more frontal activity for these responses, when compared to young listeners. Similarly, older listeners demonstrated lower amplitudes and longer latencies for omission responses than those of the younger listeners. These results are compatible with the established research on P300 and aging and suggest an effect of age on listeners’ ability to dynamically allocate attention. Correlating the P300 results with nine different speech-in-noise recognition tasks revealed a pattern of correlations indicative of different task demands in different types of speech-in-noise measures. Although fewer older participants made it difficult to look at these two age groups separately, a composite measure of speech understanding in noise, based on the nine speech recognition tasks, was found to have a positive correlation with P300 amplitude, but only in the multi-talker background condition. P300 latency was not significantly correlated with the composite speech measure, although there was a correlation with QSIN in the multi-talker condition. These results support the conclusion that the amplitude of P300 responses elicited by CV syllables presented isochronously in an oddball paradigm with background noise provides a measure of dynamic attending that is a strong predictor of speech-in-noise understanding. Combined with the differences between young and older listeners’ P300 responses, the results highlight the relevance of attentional abilities among older listeners and their possible connection to age-related differences in speech-in-noise understanding.

## Data Availability

The raw data supporting the conclusions of this article will be made available by the authors, without undue reservation.
